# First Report of a Known Pathogenic Variant in the *FZD6* Gene, in an Iranian Family with Recessive Nail Dysplasia: A Case Report

**Published:** 2019-07

**Authors:** Mirsajjad MOUSAVI-ASL GERMEH CHESHMEH, Ali NAJIZADEH, Saied HOSSEINI-ASL, Hooshang ZAIMKOHAN, Roshanak JAZAYERI

**Affiliations:** 1. Student Research Committee, Ardabil University of Medical Sciences, Ardabil, Iran; 2. Student Research Committee, Alborz University of Medical Sciences, Karaj, Iran; 3. Homa Gene Clinic, Department of Genetics, School of Medicine, Ardabil University of Medical Sciences, Ardabil, Iran; 4. Non-Communicable Diseases Research Center, Alborz University of Medical Sciences, Karaj, Iran; 5. Department of Biochemistry, Genetics and Nutrition, Faculty of Medicine, Alborz University of Medical Sciences, Karaj, Iran

**Keywords:** Nail dysplasia, *FZD6* gene, Autosomal recessive, Iran

## Abstract

Congenital Nail abnormalities are rare ectodermal defects. Autosomal recessive nail dysplasia is much rarer. Recently it has been recognized as a condition resulting in nail dystrophy in the absence of other cutaneous or extracutaneous disorders. Few case reports have identified mutations in the Frizzled 6 (*FZD6*) gene in families presenting with abnormal nails consistent with Non-Syndromic Congenital Nail Dysplasia. We report a family presenting, they lived in Namin a country of the Ardabil Province, northwestern Iran in 2016, for the first time in Iran in whom we identified mutations in *FZD6* with abnormal nails formation.

## Introduction

Congenital Nail abnormalities are rare ectodermal defects. Including very rare conditions nail dysplasia. These defects may show no significant changes or complete absence of nails (1,2).

Wnt–FZD signaling pathway plays an important role in the development of ectodermal appendages including nails. Mutations in the gene Frizzled 6 (*FZD6*) can cause autosomal-recessive nail dysplasia (2–4). We have recently identified clinical presentation of the nail disorder with a mutation in the gene frizzled causing isolated nail dysplasia in a Turkish family from Namin a country of the Ardabil Province, northwestern Iran. Some people in this area have nail dysplasia where all fingernails and toenails were claw-shaped and thickened from childhood.

This report describes a previously known missense mutation in the mentioned gene in an Iranian family, a condition reported for the first time in Iran.

## Case Report

**Fig. 1:**
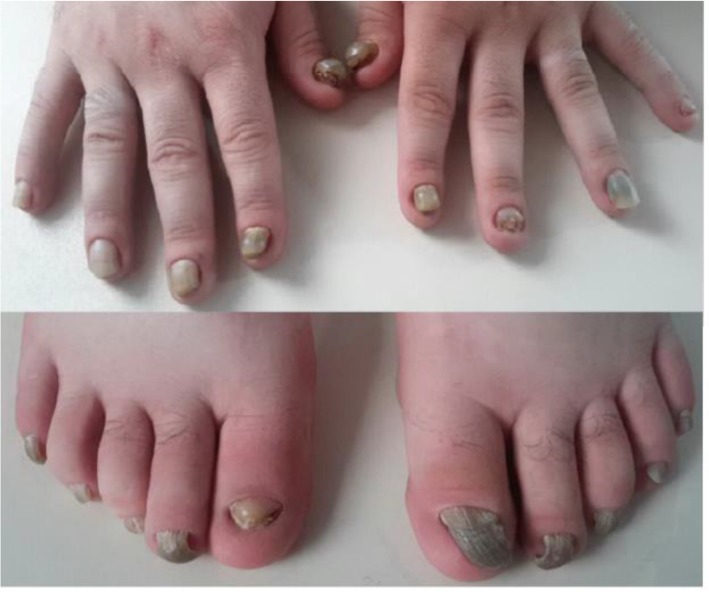
Hands and feet of proband showing that abnormality and claw-shaped in both finger and toenails at the age of 26 old years

A 26-yr-old male medical student of Ardabil University of Medical Sciences in 2016, born of a healthy non-consanguineous parent’s but the same region, referred to the genetic clinic with nail dysplasia. On clinical examination, all of the 20-nail was a variable degree of nail dystrophy, hyperkeratosis onychauxis (thick nails), hyponychia, onycholysis and claw-appearance with yellow bed (Fig. 1).

From the age of about 1 year; his nails became thickened, claw-shaped and hyperplastic but did not seem to grow out. The growing of toenails was very slower than fingernails. At first, his thumb of hands was thicker, hyperplastic and discolored and then all of the nails had symmetrical involvement the same sign. Having dealt with the surrounding surfaces deformed nail was cracked at the cuticle and extremely painful and bleeding, then separated from the nail bed and fall.

The hyperkeratotic nails developed into claw-like structures on completion of the first decade of life possibly because of an outgrowth from below the edge of the nail. No associated abnormality was found in the affected individual in his hair, teeth, skin, and scalp.

Since four years ago, he has had numerous medical problems. Such as GERD, lymphedematous on the left leg, rhinitis allergic, Lung CT Scan showed that he had allergic asthma, and abdominal ultrasonography revealed fatty liver changes in grade 4.

**Fig. 2:**
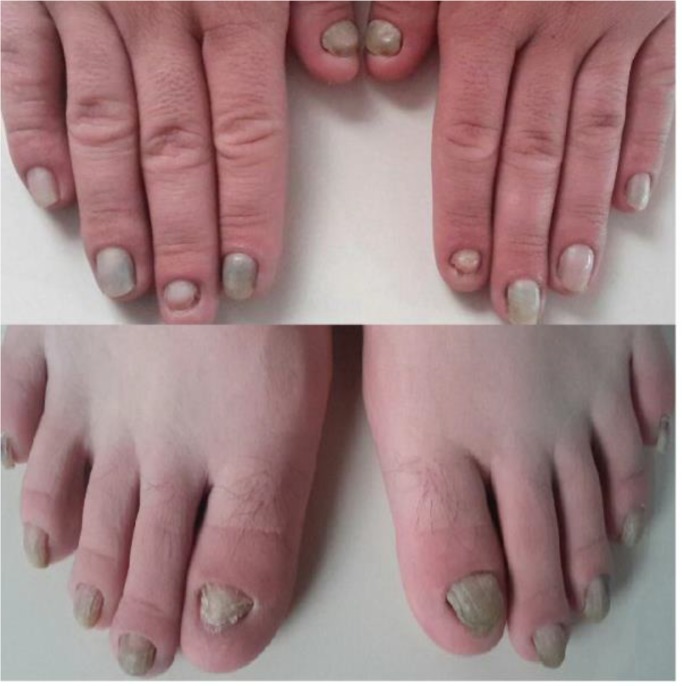
Hands and feet of the affected brother at the age of 20 year

His sister and brother were 21 and 20 yr old. His sister is healthy but his brother has the same signs and symptoms in his toenails and fingernails (Fig. 2). All observed nail abnormalities were a similar pattern of ticking and detachment of the nail from the nail bed of fingernails and toenails in both boys of this family. He had 16 aunt and uncles with no problem in their nails.

He is married and his non-consanguineous wife from another country of the Ardabil Province. She was healthy and they had no child.

Genomic DNA was obtained from blood samples from the proband. We suggested him to test his family.

Informed consent was obtained from his family and this study complied with the Declaration of Helsinki Principles. Clinical information and dermatologic examination were obtained for his family members with particular attention to nails. Permission to undertake the study was obtained from the Ethics Committee of Alborz University of Medical Sciences (Ethics Code:1395103), Karaj, Iran. Written informed consent was obtained from all patients.

### Mutation detection

5 ml venous blood was obtained from the proband and was collected in EDTA-coated tubes. The genomic DNA was extracted using the Nucleospin® DNA extraction kit (Macherey-Nagel, Düren, Germany). All seven coding exons and flanking splicing junctions of the candidate *FZD6* gene associated with autosomal recessive non-syndromic congenital Nail Dysplasia was amplified using polymerase chain reaction (PCR), using the primers listed in Table 1.

**Table 1:** Polymerase chain reaction primers for specific amplification of FZD6 gene

***Exon***	***Primer sequence***		***Tm***
2	Forward:5′- GGGGATCTTCTGAGGATGCAA-3′	Reverse: 5′-ACACAACTTGAAGAAATCGGCT-3′	58°
3	Forward: 5′-AGTTCATAAGTCTGATAGAGGGAGA-3′	Reverse: 5′-CCAGAGAGCTAGTACCAGTAGTAAA-3′	62°
4-1	Forward: 5′-TGCATTTTCATGTTCACCTGCT-3′	Reverse: 5′-TGCTCGATGGCTTCACAACT-3′	60°
4-2	Forward: 5′-TGTGCAACTCTGTTCACATTCC-3′	Reverse: 5′-TGGCTCTTGTATTTTCTCACCT-3′	60°
5	Forward: 5′-TCCCCTTTCTAGAGATTTGGTACA-3′	Reverse: 5′-ACATTGATTTGAACAGTTCCTTGGT-3′	62°
6	Forward: 5′-TCTTGCCTTAATTTCTTGCCAGT-3′	Reverse: 5′-AGGCTGAACCCAAACTTCCT-3′	58°
7	Forward: 5′-TGGACACTGGTTAGGGGTGA-3′	Reverse: 5′-ACAGTGCATAGGTCACTTCCA-3′	62°

Exon 4 was amplified into two overlapping parts so as to have shorter fragments. Each reaction mixture (25 μl) contained 20 ng genomic DNA, 1X PCR buffer, 1.5 mM MgCl_2_, 0.2 mM dNTPs, 0.5 *μ*M forward primer, 0.5 *μ*M reverse primer and 2.5 Units Taq DNA polymerase (Qiagen, Mississauga, ON, Canada).

The following PCR program was used for DNA amplification: 95 °C for 5 min; followed by 35 cycles at 95 °C for 30 sec, 57–63 °C for 30 sec (annealing temperature difference according to primer), 72 °C for 30 sec, and a final extension at 72 °C for 10 min. The PCR product of the proband was sequenced using the ABI PRISM Big Dye Terminator Cycle Sequencing Kit and ABI PRISM 3130 Genetic Analyzer (AB Applied Biosystems Veriti (96 well thermal cycler)). Alleles were discriminated using Codon Code Aligner software.

**Fig. 3:**
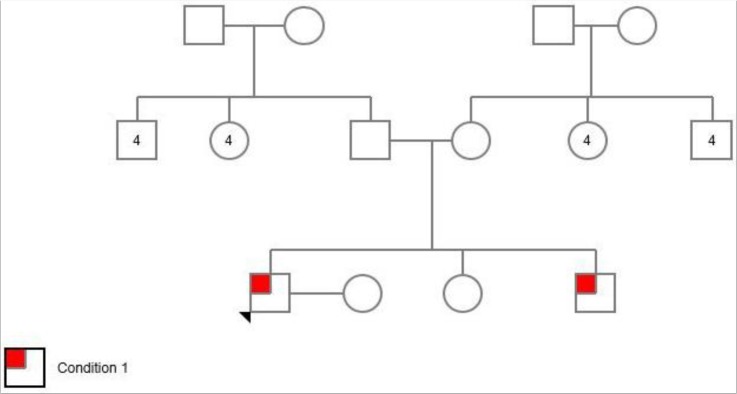
Pedigrees of families analyzed with an autosomal-recessive inherited form of isolated nail dysplasia in this study. Circles and squares represent women and men He has 16 healthy aunt and uncles

Pedigree of this family was shown in Fig. 3.

**Fig. 4:**
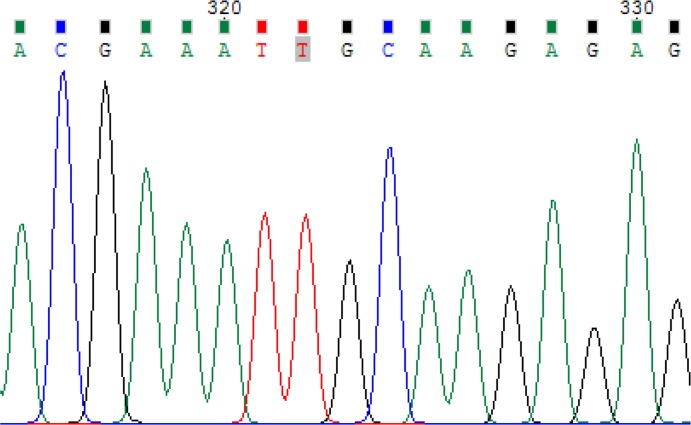
Homozygous for the FZD6 Mutations Associated with Autosomal-Recessive Nail Dysplasia (proband)

**Fig. 5:**
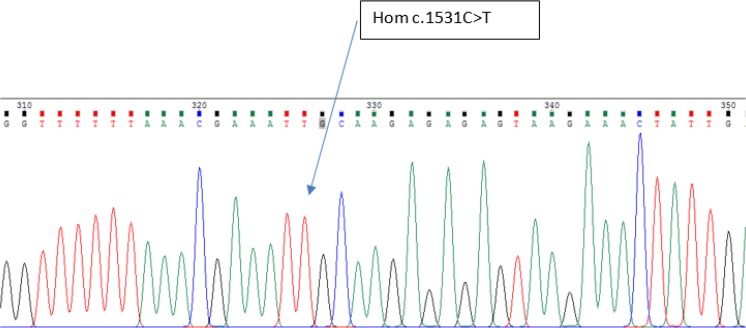
Homozygous for the FZD6 Mutations Associated with Autosomal-Recessive Nail Dysplasia (His brother: HGC339)

**Fig. 6:**
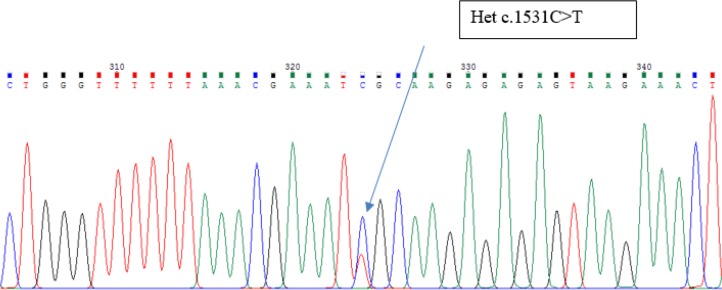
His unaffected father was heterozygous for the mutation FDZ6 (Father: HGC338)

Circles and squares represent females and males, respectively. Clear symbols represent unaffected and filled symbols represent affected individuals.After the gene analyzed, the proband was homozygous for *FZD6* mutation c.1531C>T (p. Arg511Cys) (Fig. 4). In addition, siblings and parents were also sequenced, to confirm appropriate co-segregation of the allele within the family. His younger affected brother was homozygous for the *FZD6* mutation (Fig. 5). His unaffected parents and sister were all heterozygous for the mutation (Fig. 6).

## Discussion

WNT-FZD signaling is important for the deformation of ectodermal appendages, like claw-shaped nails. It belongs to the Frizzled gene family members that serve as receptors for Wnt signaling proteins. It was mapped on chromosome 8q22.3 (2, 5–8).

Sequence analysis of *FZD6*, as the most promising gene, identified a homozygous missense mutation, in exon 4 of the gene *FZD6* in both affected individuals of our family.

This missense mutation is concordant with S.I. Raza report, identified for the first time, all with Pakistani origin (2, 7).

The missense mutation involves a C to T transition at nucleotide position 1531(c.1531C>T) substituting Arginine with Cysteine at amino acid position 511 (p. Arg511Cys). The two patients were homozygous for variant p. Arg511Cys, their parents and their normal sister were heterozygous, thus the variant co-segregated with the disease phenotype. This is an already known mutation reported for the fourth time in the world and the first time in Iran. The C>T transition changes the codon CGC (encoding arginine) to TGC (encoding cysteine) “rs number 151339003”. S.I. Raza reported the same mutation has been previously in another Pakistani family (5, 7).

All proband had a variable degree of nail dysplasia and them homozygous for carrying mutation *FDZ6* (1, 2, 8–10). During normal nail formation, the nail plate must be properly attached to the nail bed. Keratins constitute a major component of the cytoskeleton of the differentiated nail plate, and expression of *FZD6* in the nail plate corresponded to the figure and shape of affected individuals (3, 11).

The missense mutation (p. Arg511Cys) was identified in the present study, lies in the sixth transmembrane domain of the mutant *FZD6*. Considering the effect of the missense mutation (p. Arg511Cys) described the mutation (p. Arg511Cys) lies in the transmembrane domain, it is possible that mutant *FZD6* reaches to the plasma membrane and can affect the binding of Wnt ligand with *FZD6*, which in turn will affect recruitment of intracellular protein.

Dermatologists should consider the genetic analysis of individuals with hereditary isolated nail dysplasia, which aids in correct genetic counseling for their family.

## Ethical considerations

Ethical issues (Including plagiarism, informed consent, misconduct, data fabrication and/or falsification, double publication and/or submission, redundancy, etc.) have been completely observed by the authors.

## References

[1] Wilson NJ Hansen CD Azkur D (2013). Recessive mutations in the gene encoding frizzled 6 cause twenty nail dystrophy--expanding the differential diagnosis for pachyonychia congenita. J Dermatol Sci, 70(1):58–60. 23374899 10.1016/j.jdermsci.2012.12.005

[2] Frojmark AS Schuster J Sobol M (2011 ) Mutations in Frizzled 6 cause isolated autosomal-recessive nail dysplasia. Am J Hum Genet, 88(6):852–60. 21665003 10.1016/j.ajhg.2011.05.013 PMC3113248

[3] Cui CY Klar J Georgii-Heming P (2013). Frizzled6 deficiency disrupts the differentiation process of nail development. J Invest Dermatol, 133(8):1990–7. 23439395 10.1038/jid.2013.84 PMC3695035

[4] De Marco P Merello E Rossi A (2012). FZD6 is a novel gene for human neural tube defects. Hum Mutat, 33(2):384–90. 22045688 10.1002/humu.21643 PMC3482927

[5] Khan S Ansar M Kamal Khan A (2018). A Homozygous Missense Mutation in SLC25A16 is Associated with Autosomal Recessive Isolated Fingernail Dysplasia in a Pakistani Family. Br J Dermatol, 178(2): 556–558. 28504827 10.1111/bjd.15661 PMC5685937

[6] Kasparis C Reid D Wilson NJ (2016). Isolated recessive nail dysplasia caused by FZD6 mutations: report of three families and review of the literature. Clin Exp Dermatol, 41(8):884–9. 27786367 10.1111/ced.12934 PMC5132090

[7] Raza SI Muhammad N Khan S Ahmad W (2013). A novel missense mutation in the gene FZD6 underlies autosomal recessive nail dysplasia. Br J Dermatol, 168(2):422–5. 22861124 10.1111/j.1365-2133.2012.11203.x

[8] Naz G Pasternack SM Perrin C (2012). FZD6 encoding the Wnt receptor frizzled 6 is mutated in autosomal-recessive nail dysplasia. Br J Dermatol, 166(5):1088–94. 22211385 10.1111/j.1365-2133.2011.10800.x

[9] Kilander MB Dahlstrom J Schulte G (2014). Assessment of Frizzled 6 membrane mobility by FRAP supports G protein coupling and reveals WNT-Frizzled selectivity. Cell Signal, 26(9):1943–9. 24873871 10.1016/j.cellsig.2014.05.012

[10] Khan S Basit S Habib R Kamal A Muhammad N Ahmad W (2015). Genetics of human isolated hereditary nail disorders. Br J Dermatol, 173(4):922–9. 26149975 10.1111/bjd.14023

[11] Krebsova A Hamm H Karl S Reis A Hennies HC (2000) Assignment of the gene for a new hereditary nail disorder, isolated congenital nail dysplasia, to chromosome 17p13. J Invest Dermatol, 115(4):664–7. 10998140 10.1046/j.1523-1747.2000.00102.x

